# Circadian Regulation of Bone Remodeling

**DOI:** 10.3390/ijms25094717

**Published:** 2024-04-26

**Authors:** Nobuaki Kikyo

**Affiliations:** 1 Stem Cell Institute, Minneapolis, MN 55455, USA; kikyo001@umn.edu; 2 Department of Genetics, Cell Biology, and Development, University of Minnesota, Minneapolis, MN 55455, USA

**Keywords:** *Bmal1*, bone, bone remodeling, bone turnover marker, circadian rhythms, M-CSF, osteoblast, osteoclast, osteocyte, RANKL

## Abstract

Adult bones are continuously remodeled by the balance between bone resorption by osteoclasts and subsequent bone formation by osteoblasts. Many studies have provided molecular evidence that bone remodeling is under the control of circadian rhythms. Circadian fluctuations have been reported in the serum and urine levels of bone turnover markers, such as digested collagen fragments and bone alkaline phosphatase. Additionally, the expressions of over a quarter of all transcripts in bones show circadian rhythmicity, including the genes encoding master transcription factors for osteoblastogenesis and osteoclastogenesis, osteogenic cytokines, and signaling pathway proteins. Serum levels of calcium, phosphate, parathyroid hormone, and calcitonin also display circadian rhythmicity. Finally, osteoblast- and osteoclast-specific knockout mice targeting the core circadian regulator gene *Bmal1* show disrupted bone remodeling, although the results have not always been consistent. Despite these studies, however, establishing a direct link between circadian rhythms and bone remodeling in vivo remains a major challenge. It is nearly impossible to repeatedly collect bone materials from human subjects while following circadian changes. In addition, the differences in circadian gene regulation between diurnal humans and nocturnal mice, the main model organism, remain unclear. Filling the knowledge gap in the circadian regulation of bone remodeling could reveal novel regulatory mechanisms underlying many bone disorders including osteoporosis, genetic diseases, and fracture healing. This is also an important question for the basic understanding of how cell differentiation progresses under the influence of cyclically fluctuating environments.

## 1. Introduction

Bones provide frameworks for animal body structure and movement, calcium and phosphate reservoirs, cavities for hematopoiesis, and protective shells for vital organs such as the brain (skull), the heart and lungs (ribs and sternum), and the spinal cord (vertebrae). Bone components are continuously renewed through the activities of two competing cell types: bone-resorbing osteoclasts and bone-forming osteoblasts [1,2,3,4,5]. Osteoblasts secrete type I collagen and other extracellular matrix (ECM) proteins, providing a scaffold for the subsequent precipitation of hydroxyapatite (Ca_10_(PO_4_)_6_(OH)_2_). In contrast, multi-nucleated phagocytotic osteoclasts dissolve hydroxyapatite with secreted H^+^ and Cl^−^ ions and digest the ECM proteins with proteases, such as cathepsin K and matrix metalloproteinases (MMPs). These contrasting activities are controlled by stroma cells, immune cells, and circulating hormones, in addition to the crosstalk between osteoblasts, osteocytes differentiated from osteoblasts, and osteoclasts.

Bones undergo two types of turnover called bone remodeling and bone modeling [6,7,8,9,10]. Bone remodeling replaces old bones with new bones without changing the bone shape or size in response to daily micro-damages. Bone remodeling also ensures the maintenance of calcium homeostasis during adult life. An essential feature of bone remodeling is the sequential activity of bone resorption by osteoclasts, followed by bone formation by osteoblasts at the same location on the surface of or inside (Haversian systems) the bones, coordinating as basic multicellular units (BMUs). On the other hand, bone modeling is characterized by changes in bone shape via independent activities of osteoblasts and osteoclasts in terms of time and space in the bone without forming BMUs. Bone modeling mainly occurs during bone growth, but it continues throughout life. Since human studies of circadian bone biology primarily use adult subjects, the results primarily reflect bone remodeling. In contrast, mouse circadian studies can cover bone modeling during the development of knockout mice, in addition to bone remodeling in adult mice.

Circadian rhythms are organized via two ubiquitous feedback loops centered on the heterodimers of the circadian locomotor output cycles protein kaput (CLOCK) and brain and muscle ARNT-like 1 (BMAL1) proteins. Several approaches have shown that bone remodeling is under the control of circadian regulators [11,12,13,14,15]. However, human data are almost limited to the circadian measurement of bone turnover markers (BTMs), hormones, and minerals (calcium and phosphate) in the blood and urine. Gene expression analyses have mainly come from mouse models, including transcriptome analyses and knockout mice. Despite the difficulty in connecting human circadian rhythms and bones at the molecular level, various types of disruption to circadian rhythms result in human bone pathology. The most extensively studied examples include osteoporosis related to shift work and sleep deprivation, although it has been pointed out that general lifestyle changes (e.g., eating time and decreased exercise) could also be responsible [13].

The first part of the current article will introduce information on the cellular and protein players of bone remodeling and then outline circadian gene regulation. The second part will discuss the molecular evidence primarily connecting bone remodeling and circadian rhythms. The contrast between the first and second parts highlights the limited understanding we have regarding how circadian rhythms regulate the molecular mechanisms involved in bone remodeling cells, cytokine signaling, and the secretion of ECM proteins, enzymes, H^+^, and Cl^−^ in vivo. These details are important for a deeper understanding of physiological bone remodeling, as well as the development of interventions for various bone diseases. Studies of circadian gene regulation are commonly linked to chronotherapy (the administration of therapeutic agents at a specific time of day); however, this is not discussed in the current article because the main goal is to highlight the gap between observed circadian-related changes in bone and what we know about the details of bone remodeling mechanisms. Readers are referred to recent review articles for more information on bone chronotherapy [13,16].

## 2. Differentiation and Functions of Osteoblasts, Osteocytes, and Osteoclasts

Embryonic osteogenic cells are derived from different tissues depending on the location of the bones [17,18,19]. Osteoblasts originating from the paraxial mesoderm form the dorsal cranial bones and axial skeleton (e.g., vertebrae, sacrum, and rib cage), whereas those derived from the lateral plate mesoderm contribute to the appendicular skeleton (e.g., upper and lower extremities, hands, feet, shoulder girdle, and pelvis). In addition, the frontal cranium and facial bones are formed by neural crest cells originating from the ectoderm. Postnatal osteoblasts, which are more relevant to the current article, are derived from mesenchymal stem cells (MSCs) in the bone marrow [1,2,20]. MSCs first differentiate into chondro-osteoprogenitor cells, which sequentially differentiate into osteoprogenitor cells, preosteoblasts, and, finally, osteoblasts by activating the osteogenic master transcription factor genes runt-related transcription factor 2 (*Runx2*) and Osterix (*Osx*). These and other transcription factors drive osteoblast differentiation in coordination with several other signaling pathways driven via bone morphogenetic proteins (BMPs), transforming growth factor β (TGF-β), wingless-related integration site (Wnt/β-catenin, and sonic hedgehog, among others [21]. Osteoblasts ultimately have one of three cell fates: they differentiate into quiescent bone lining cells on the bone surface, terminally differentiate into osteocytes entombed in the space called lacunae within the mineralized bone matrix, or undergo apoptosis [22].

### 2.1. Osteoblasts

Osteoblasts play a central role in bone formation through two major activities: building a dense scaffold by secreting extracellular matrix (ECM) proteins and providing rigidity to the scaffold through mineralization [1,2]. Osteoblasts secrete type I collagen as the most abundant bone ECM protein, comprising around 10% of the dry mass of bone. Two α1 procollagen chains (encoded by the *Col1a1* gene) and one α2 chain (*Col1a2*) are self-assembled into a triple helix, which are then aligned side-by-side and longitudinally, cross-linked, and ultimately built into collagen fibers [23,24]. Collagen provides tensile strength (flexibility) to bone, as well as a scaffold for the deposition of minerals, primarily hydroxyapatite in the intra- and inter-collagen spaces. Hydroxyapatite comprises around 90% of the bone matrix and is generated by the precipitation of calcium ions (Ca^2+^) and phosphate ions (PO_4_^−^) exported from osteoblasts into the extracellular space [25,26]. In addition, osteoblasts promote mineralization by secreting alkaline phosphatase (ALP), which remains attached to the plasma membrane and hydrolyzes pyrophosphate (P_2_O_7_, an inhibitor of hydroxyapatite formation [27]) in the extracellular space. Moreover, the generation of inorganic phosphorous (Pi) from pyrophosphate via hydroxylation increases the supply of Pi and further promotes the deposition of hydroxyapatite. Hydroxyapatite formation is also affected by the concentrations of Ca^2+^ and PO_4_^−^ in the extracellular space, reflecting the circulating levels under the control of the parathyroid hormone (PTH) and vitamin D (see below for more details).

### 2.2. Osteocytes

Between 5 and 20% of osteoblasts follow the terminal differentiation pathway to become osteocytes, which comprise around 90–95% of the cell population in adult mammalian bone [28]. In contrast, osteocytes are completely missing in most boney fishes, which constitute about half of vertebrates [29]. Although osteocytes are embedded within the mineralized matrix, they are far from isolated or inactive [30,31,32]. They extend cytoplasmic processes to nearby osteocytes, osteoblasts, and bone lining cells through the canaliculi—narrow passages preserved during mineralization—creating vast networks linked via gap junctions. Osteocytes are tightly integrated into the regulatory network of bone remodeling and calcium–phosphate homeostasis, as discussed below. The synthesis of ECM proteins is downregulated and the proteins necessary for bone remodeling signaling are upregulated in osteocytes compared with osteoblasts.

### 2.3. Bone Lining Cells

Although bone lining cells have rarely been studied in circadian bone research, they play important roles during bone remodeling [33,34]. For example, MMPs secreted by bone lining cells eliminate the remaining non-mineralized matrix on the surface of the mineralized bones prior to resorption by osteoclasts [33,35]. This is a necessary step for bone resorption because osteoclasts do not bind the non-mineralized matrix. In addition, bone lining cells regulate osteoclastogenesis through the secretion of the receptor activator of nuclear factor κB ligand (RANKL) and osteoprotegerin (OPG) [34].

### 2.4. Osteoclasts

In contrast to osteoblasts, osteoclasts belong to the hematopoietic system and are generated via the fusion of multiple osteoclast progenitor cells derived from the monocyte and macrophage lineage [3,5,36]. The main function of osteoclasts is osteolysis, which is primarily achieved via two activities [35]. First, osteoclasts secrete H^+^ and Cl^−^ to acidify and dissolve hydroxyapatite. Second, osteoclasts digest type I collagen and other ECM proteins with secreted proteases, such as cathepsin K and MMPs, such as MMP9 and MMP14. The digestion by cathepsin K is promoted by the acidic environment. The two most important factors for osteoclastogenesis are macrophage colony-stimulating factor (M-CSF) and RANKL secreted by osteoblasts, osteocytes, and other cells.

## 3. Interactions between Osteoblasts, Osteocytes, and Osteoclasts during Bone Remodeling

Although bone remodeling is regulated by many systemic factors, including hormones, diet, weight bearing, and sleep cycles, these factors ultimately converge to place osteoblasts, osteocytes, bone lining cells, and osteoclasts as the frontline effectors. The activities and numbers of these cells are modulated by crosstalk between these cells, as summarized in several recent review articles [3,9,37,38]. These review articles listed more than 20 examples of crosstalk molecules in total. However, many interpretations of crosstalk are based on co-culture and other in vitro experiments; some of them have not been confirmed by or are inconsistent with mouse-based in vivo results, as previously pointed out in a critical review article [38]. Nonetheless, given the difficulty in obtaining pure and sufficient cells from bone, in vitro data have been instrumental in guiding what to look for in in vivo models. The following sections discuss the exemplary crosstalk established in vitro and corroborated in vivo. This includes crosstalk in the direction from osteoblasts and osteocytes to osteoclasts (RANKL and M-CSF), in the opposite direction (the Semaphorin 4D–Plexin-B1 interaction), and between osteocytes and osteoblasts (sclerostin and DKK1).

### 3.1. RANKL and RANK

One of the best-characterized crosstalk examples is mediated by RANKL (produced by osteoblasts, osteocytes, and bone lining cells) and the receptor RANK (receptor activator of nuclear factor κB) on the surface of osteoclast progenitors and osteoclasts [39,40]. RANKL belongs to the tumor necrosis factor (TNF) superfamily and is present in two active forms: a transmembrane form and a cleaved soluble form composed of the extracellular domain alone. The interaction between RANKL and RANK eventually leads to the expression of the nuclear factor of the activated T cells 1 (*Nfatc1*) gene as the master transcription factor for osteoclastogenesis. NFATc1 activates many osteoclast-specific genes, including cathepsin K (*Ctsk*), tartrate-resistant acid phosphatase 5 (*Trap* or *Acp5*, a marker protein widely used to detect osteoclasts via enzyme histochemistry), and calcitonin receptor (*Calcr*) [41]. Osteoblasts and osteocytes also secrete OPG, which is a member of the TNF receptor superfamily but lacks a transmembrane domain. OPG sequesters RANKL as a soluble decoy receptor, inhibiting osteoclastogenesis. Thus, the bone-forming cells both activate and inhibit osteoclastogenesis through the RANK-RANKL-OPG axis in a context-dependent manner. These roles of the axis have been verified in vivo with genetically modified mouse models and via the study of various human mutations [36].

### 3.2. M-CSF

Another crucial cytokine for osteoclastogenesis is M-CSF, which is also secreted by osteoblasts and osteocytes [42,43]. M-CSF promotes the proliferation and differentiation of monocytes and macrophages, as well as osteoclast differentiation and maturation in combination with RANKL. Like RANKL, M-CSF has a membrane-bound form and a secreted form, both of which promote osteoclastogenesis through binding to its receptors on osteoclasts. The requirement for M-CSF for osteoclastogenesis in vivo has been demonstrated with *op*/*op* mutant mice (op stands for osteopetrosis, a condition characterized by abnormally dense bone formation), where the M-CSF gene (*Csf1*) is inactivated. The mice initially show osteopetrosis with reduced monocytes and macrophages when they are young, but the phenotype is subsequently compensated for by other molecules [44,45].

### 3.3. Semaphorin 4D and Plexin-B1

The interaction between the axon-guidance protein Semaphorin 4D secreted by osteoclasts and its receptor Plexin-B1 on osteoblasts is another example of crosstalk. The binding of these proteins inhibits osteoblast differentiation and motility in vitro [46]. Consistently, *Sema4d*^−/−^ mice and *Plxnb1*^−/−^ mice demonstrate an increase in bone mass. The injection of a blocking antibody against Semaphorin 4D prevented bone loss due to ovariectomy in a postmenopausal mouse model, suggesting that the Semaphorin 4D–Plexin-B1 interaction is important for the pathogenesis of postmenopausal osteoporosis.

### 3.4. Sclerostin and DKK1

Crosstalk also happens between osteocytes and osteoblasts by two secreted Wnt inhibitors, sclerostin and Dickkopf Wnt signaling pathway inhibitor 1 (DKK1), potentially as a feedback mechanism to prevent excessive bone formation. Both are abundantly secreted by osteocytes and bind the lipoprotein receptor-related protein 5 (LRP5) and LRP6 coreceptors on osteoblasts, inhibiting canonical Wnt signaling [47,48]. LRP5 and LRP6 have four β-propeller (BP) domains where a Wnt ligand binds. While sclerostin binds the BP1 domain, DKK1 occupies the BP1 and BP3 domains, both preventing the binding of a Wnt ligand. A rare human autosomal recessive disease called sclerosteosis, which is characterized by increased bone mass and cortical thickening, is caused by mutations in the sclerostin (*SOST*) gene [49]. *Sost*^−/−^ mice also demonstrate markedly increased bone mass and increased trabecular bones [50]. Similarly, the osteoblast-specific disruption of *Sost* increases bone mass and decreases osteoclasts [51].

## 4. Circadian Regulation of Gene Expression

**Figure 1:**
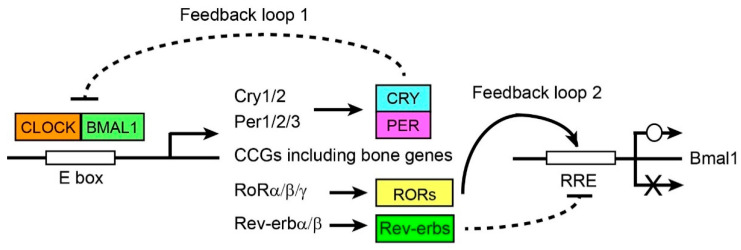
Summary of two circadian rhythm feedback loops. Abbreviations are as follows: CCGs—clock-controlled genes; RRE—Rev-erb/ROR response elements.

Circadian rhythms in the body align homeostasis to the 24 h cycle of environmental changes. In mammals, two feedback loops build robust mechanisms to maintain rhythmicity in the body (Figure 1) [52,53]. The first feedback loop is initiated by the binding of the CLOCK/BMAL1 heterodimer to the E box sequences in the promoters and enhancers of hundreds of genes (called clock-controlled genes or CCGs). CCGs encompass more than 20% of whole transcriptomes if all tissues are combined. Importantly, CCGs include cryptochromes (*Cry1* and *Cry2*) and periods (*Per1*–*Per3*), creating the repressor heterodimer CRY/PER that binds CLOCK/BMAL1 and inhibits the transcription activity. This makes the upregulation of *Cry* and *Per* transient at the mRNA level. In addition, the CRY and PER proteins undergo proteasomal degradation several hours later, which also contributes to making the upregulation of CRY/PER transient at the protein level. The downregulation of CRY and PER de-represses CLOCK/BMAL1, allowing the complex to start a new transcription cycle. In the second feedback loop, CLOCK/BMAL1 induces the transcription of the genes encoding reverse orientation c-erbAα (Rev-erbα) and Rev-erbβ as well as retinoic acid receptor-related orphan receptor α (RORα), RORβ, and RORγ. Rev-erbs inhibit and RORs activate the transcription of *Bmal1* through binding the Rev-erb/ROR response elements (RREs) in the *Bmal1* promoter. CLOCK/BMAL1-centered feedback loops exist almost ubiquitously in mammalian bodies (peripheral clocks), including in the bones [54], and they can maintain circadian rhythms autonomously. However, peripheral clocks are generally synchronized by the central clock in the suprachiasmatic nucleus in the hypothalamus, which is trained by the light signal coming from the retina.

## 5. Circadian Rhythms of Bone Turnover Markers

Molecular analyses of circadian rhythms with in vivo materials have been significantly delayed in humans compared with mice and other model species due to the difficulty in repeatedly obtaining samples from the body. A guideline for studies of circadian rhythms recommends collecting samples every 2 h to assess the presence of circadian rhythmicity [55]. A period of 4 h has been more commonly used, although it is still difficult, if not impossible, with human subjects. In this context, the measurement of serum and urine levels of bone turnover markers (BTMs) provides a rare window to assess circadian rhythmicity in bone remodeling in the human body, however imperfect this might be. BTMs have been extensively used as clinical markers of bone remodeling in osteoporosis and other bone diseases [13,16,56,57,58]. For example, the International Osteoporosis Foundation and the International Federation of Clinical Chemistry and Laboratory Medicine have recommended crosslinked C-terminal telopeptide of type I collagen (CTX) and procollagen type I N-terminal propeptide (PINP) as BTMs to assess osteoporosis [59].

BTMs can be divided into two categories: those for bone resorption and those for bone formation. BTMs representing bone resorption include serum or urine levels of various peptide fragments derived from degraded type I collagen. BTMs for bone formation are generally products secreted by osteoblasts, such as osteocalcin, bone isozyme of alkaline phosphatase (bone ALK), and PINP.

### 5.1. Circadian Rhythms of BTMs Representing Bone Resorption

#### 5.1.1. CTX

CTX is released from type I collagen by cathepsin K and other enzymes during bone resorption [56]. The serum concentration of CTX shows the clearest circadian fluctuations among all BTMs discussed here because of its high amplitude (the peak-to-average difference is 30–80% of the average value). For example, when the average and the peak serum concentrations of CTX were 0.32 ng/mL and 0.5 ng/mL, respectively, the amplitude was calculated as 56% [(0.5–0.32)/0.32] [60]. The peak (3:00–8:00) and nadir times (13:00–20:00) are surprisingly consistent among the eight reports listed in Table 1. Age, sex, and ethnicity do not affect the circadian pattern; however, fasting and vigorous physical activities can alter the pattern [61]. Yavropoulou et al. pointed out that food intake, rather than intrinsic circadian rhythms, could be responsible for the apparent circadian changes in BTMs for bone resorption [62]. As supporting evidence, several gut-derived peptides, such as glucose-dependent insulinotropic peptide (GIP), glucagon-like peptide 1 (GLP-1), and GLP-2, are induced by food intake and modulate the activities of osteoblasts and osteoclasts. Since BTMs for bone formation are not affected by food intake [62], the intrinsic clock appears to contribute to the rhythmicity of BTMs at a certain level, regardless of the roles of gut peptides.

#### 5.1.2. Crosslinked N-Terminal Telopeptide of Type I Collagen (NTX)

NTX is released from type I collagens like CTX during bone resorption, but it is commonly measured in the urine and normalized with creatinine concentrations [56]. The circadian pattern of the NTX/creatinine ratio is similar to the serum pattern of CTX (Table 1), but the amplitude is lower (<30%). However, collecting urine for the measurement of NTX has an advantage of being less invasive than repeated venipunctures for the measurement of CTX.

**Table 1:** Circadian rhythms of human BTMs for bone resorption. Serum and urine were used for CTX and NTX/cre, respectively. Peak and nadir times are described using the 24 h clock. Abbreviations are as follows: BTM—bone turnover marker; CTX—crosslinked C-terminal telopeptide of type I collagen; NTX/cre—crosslinked N-terminal telopeptide of type I collagen/creatinine ratio; M—men; and W—women.

BTM	Number and Sex of Subjects	Age (Years Old)	Peak Time	Nadir Time	Reference
CTX	M and W, total 100	Unknown	5–6	14–20	[63]
CTX	6 M and 12 W	28–71	3	18	[64]
CTX	20 W	25–65	6	18	[60]
CTX	3 ethnic groups *n* = 30 or 31 each M and W mixed	60–75	5–6	14–17	[65]
CTX	10 M	20–65	6	13–14	[66]
CTX	20 M and 18 W	52–76	6	16	[67]
CTX	9 W	19–60	8	16	[68]
CTX	5 M and 5 W	31–51	4	14–16	[69]
NTX/cre	18 W	20–47	3–7	15–19	[70]
NTX/cre	20 M and 15 W	27–71	8	19	[71]
NTX/cre	14 M and 14 W	>65	0–6	16–20	[72]
NTX/cre	15 W	20–23	8	14 or 20 (no graph)	[73]

### 5.2. Circadian Rhythms of BTMs for Bone Formation

#### 5.2.1. Osteocalcin

Osteocalcin is a multi-functional ECM protein secreted by osteoblasts; its roles include glucose metabolism, male fertility, and brain function, but its roles in bone formation have not been established [74,75]. Since there are more than ten types of osteocalcin fragments in the serum, it is recommended to measure the total osteocalcin level, including the intact protein [56,76]. The serum level reaches its peak in the early morning (1:00–6:00) and gradually reaches the nadir in the early afternoon (Table 2). Although it has been repeatedly studied, the amplitude is low (<20%), which could obscure circadian fluctuations, as reported by Hansen et al. [69].

#### 5.2.2. Bone ALP

There are four isozymes of ALP; bone ALP belongs to the tissue-nonspecific isozyme, which is expressed in the liver and kidneys, among other tissues [77]. However, post-translational modifications by N- and O-glycosylation and sialic acid can distinguish bone ALP from other tissue-nonspecific ALPs [58]. The serum level of bone ALP is also under circadian regulation; however, the peak and nadir times vary depending on the study. While Greenspan et al. detected the peak at noon and the nadir in the afternoon [72], three other groups reported variable results among subjects or a lack of rhythmicity (Table 2) [65,69,73]. A low amplitude (<15%) seems to be a fundamental contributing factor for the differences among the studies, in addition to the differences in the subject conditions.

#### 5.2.3. PINP

N- and C-propeptidases cleave the secreted procollagen triple helix to release N- and C-terminal propeptides, respectively, in the ECM [24]. All subjects in the study by Redmond et al. [65] and men in the report by van der Spoel et al. [67] showed the circadian rhythmicity of PINP, with the serum peak level between 2:00 and 4:00 and the nadir between 9:00 and 12:00, but the amplitude was <10%. However, Swanson et al.’s study [66] and women in the study by van der Spoel et al. [67] did not show any rhythmicity. The variability in these results could also stem from the low amplitude.

### 5.3. Interpretations of Circadian Expression of BTMs

In summary, CTX displays the highest amplitude; the amplitude of the other BTMs ranges from modest (<30%) to very low (<10%). Although the rhythmicity was found to be statistically significant, the physiological significance of rhythmicity with low amplitude is not known. Even for CTX, the time of the peak level may not directly represent the time of the maximum bone resorption due to a lag period before entering circulation, food intake, or other influential factors. To establish a tighter circadian connection between serum CTX concentration and local bone resorption level, a direct comparison between these parameters with animal models would be required.

**Table 2:** Circadian rhythms of human markers for bone resorption. Serum was used for all BTMs. Peak and nadir times are described using the 24 h clock. Abbreviations are as follows: BTM—bone turnover marker; ALP—alkaline phosphatase; PINP—procollagen type I N-terminal propeptide; M—men; and W—women.

BTM	Number and Sex of Subjects	Age (Years Old)	Peak Time	Nadir Time	Reference
Osteocalcin	6 M and 4 W	20–30	4	12	[78]
Osteocalcin	14 M and 14 W	>65	M 2 and W 20	M 15 and W 12	[72]
Osteocalcin	20 W	25–65	6	18	[60]
Osteocalcin	3 ethnic groups *n* = 30 or 31 each M and W mixed	60–75	1–5	9–13	[65]
Osteocalcin	20 M and 18 W	52–76	2	14	[67]
Osteocalcin	9 W	19–60	6	14	[68]
Osteocalcin	5 M and 5W	31–51	No rhythmicity		[69]
Bone ALP	14 M and 14 W	>65	12	2–6	[72]
Bone ALP	15 W	20–23	Same at 8, 14, and 20	Same at 8, 14, and 20	[73]
Bone ALP	3 ethnic groups *n* = 30 or 31 each M and W mixed	60–75	11–20 variable	4	[65]
Bone ALP	5 M and 5W	31–51	No rhythmicity		[69]
PINP	3 ethnic groups *n* = 30 or 31 each M and W mixed	60–75	2–4	9–12	[65]
PINP	20 M and 18 W	52–76	M 2 and W flat	M 10 and W flat	[67]
PINP	10 M	20–65	No rhythmicity		[66]

## 6. Circadian Rhythms of Gene Expression Relevant to Bone Remodeling

More direct evidence for the circadian regulation of bone activities has come from the expression analyses of osteoblast and osteoclast genes in mouse models. This includes a genome-wide transcriptome analysis and PCR-based quantification of specific genes (Table 3). Circadian changes in the levels of PTH, calcitonin (CT), vitamin D, Ca^2+^, and phosphate are also included in this section as relevant regulators of bone remodeling.

### 6.1. Circadian Expression of Bone Genes

In a widely cited microarray study, Zvonic et al. found that 26% of all genes showed circadian expression in mouse calvaria [54]. The oscillating genes included those encoding cytokines (BMPs, fibroblast growth factors (FGFs), TGF-βs, and M-CSF), signaling proteins and transcription factors (RUNX1-3, STAT, NF-κB, and Frizzled), and bone-specific functional/structural proteins (collagen, calcium channels, and ALP), in addition to circadian core regulators (BMAL1, CRYs, and PERs). Other groups studied the circadian expression of specific genes in the tibia or femur using quantitative PCR. Circadian time is commonly designated as zeitgeber (time cue) time or ZT in these experiments; lights are on between ZT0 and ZT12 and off between ZT12 and ZT24. For example, if the light is turned on at 6:00 and off at 18:00, 6:00 and 18:00 correspond to ZT0 and ZT12, respectively. Mouse studies showed that the expression of representative genes for osteoblasts (*Runx2* and *Tnfrsf11b* encoding OPG) and osteoclasts (*Nfatc1*, *Tnfsf11* encoding RANKL, and *Ctsk* encoding cathepsin K) was under circadian regulation (Table 3). This list includes master transcription factors for osteoblasts (*Runx2*) and osteoclasts (*Nfatc1*), but it is not known whether their entire differentiation programs, composed of hundreds of downstream genes, progress in a circadian-dependent manner.

**Table 3:** Circadian expression of mouse genes in bone. Mice were housed under a 12 h light and 12 h dark cycle. Abbreviations are as follows: *Runx2*—runt-related transcription factor 2; *Tnfrsf11b*—tumor necrosis factor receptor superfamily, member 11; *Tnfsf11*—tumor necrosis factor superfamily, member 11; *Nfatc1*—nuclear factor of activated T cells 1; *Ctsk*—cathepsin K; and ZT—zeitgeber time.

Gene and Protein	Source	Peak Time	Nadir Time	References
*Runx2*	Calvaria	Circadian rhythms exist but no detail given		[54]
*Runx2*	Calvaria or tibia	ZT0 and ZT14	ZT10	[79]
*Tnfrsf11b* (*Opg*)	Tibia	ZT12	ZT0	[80]
*Tnfsf11* (*Rankl*)	Tibia	ZT20	ZT9	[80]
*Nfatc1*	Femur	ZT12	ZT20-ZT24	[81]
*Nfatc1*	Tibia and femur	ZT8	ZT0-ZT12	[82]
*Ctsk*	Femur	ZT12-ZT16	ZT18-ZT8	[81]
*Ctsk*	Tibia	ZT20	ZT9	[80]

### 6.2. Calcium Metabolism via PTH, Vitamin D, and Calcitonin

Bones contain 99% of all calcium in the body as hydroxyapatite, but it is the ionic form of calcium (Ca^2+^) in serum that is the main target of regulation by three hormones; specifically, it is increased by PTH and vitamin D and decreased by CT (mainly in mice) [83,84,85,86,87]. The circadian expression of PTH has been well established in humans, unlike the two other hormones. PTH raises serum Ca^2+^ concentration by increasing bone resorption (through upregulated RANKL in osteoblasts), Ca^2+^ resorption by renal tubules, and Ca^2+^ absorption from the intestines (through increased vitamin D synthesis in the kidneys). The serum PTH level is generally high in the early morning or evening and low during the daytime, with an amplitude of 10–60% (Table 4). The circadian pattern of the serum Ca^2+^ concentration is roughly anti-phasic to the PTH pattern (Table 4), but many other factors are likely to be involved in determining the Ca^2+^ pattern.

Vitamin D precursors are taken up from food or synthesized by skin cells, which are catalyzed via UV radiation from sunlight. They are subsequently metabolized in the liver and finally converted to two active forms: 1,25(OH)_2_D_3_ and 24,25(OH)_2_D_3_. Both forms raise serum Ca^2+^ concentration by increasing absorption from the intestines, bone resorption by osteoclasts, and PTH activity in the renal tubules [83,86,88]. Due to its long half-life (6–8 h) [83] and its synthesis by sunlight, the intrinsic circadian rhythms at the serum level of vitamin D are not clear.

The third hormone, CT, is secreted by parafollicular cells in the thyroid gland in response to elevated serum Ca^2+^ concentrations. It reduces serum Ca^2+^ by inhibiting osteoclast activity and decreasing Ca^2+^ resorption by the renal tubules in mice [83,84]. However, the role of CT in human Ca^2+^ regulation remains unclear. The serum CT level shows circadian oscillations with bimodal peaks with an amplitude of around 50% (Table 4).

### 6.3. Serum Phosphate

Around 85% of the total phosphorous in the body exists as hydroxyapatite in bones; the remaining phosphorous is bound to proteins and other organic molecules or remains as inorganic phosphate (Pi) [89]. Pi in serum mainly exists as phosphate ions (PO_4_^3−^) and its concentration shows circadian rhythms with an amplitude of <25% (Table 4). The serum Pi concentration is primarily regulated by three soluble factors: PTH, vitamin D, and FGF23 [89,90]. The regulation of Pi by PTH is more complicated than the regulation of Ca^2+^. While PTH increases Pi levels along with Ca^2+^ by increasing bone resorption, it decreases Pi levels by promoting renal excretion. Vitamin D raises the serum Pi level by increasing intestinal absorption and renal reabsorption [90]. FGF23, secreted by osteocytes, reduces renal Pi resorption, but its circadian rhythmicity remains unclear. In summary, although only PTH is known to show circadian rhythms in serum concentration among the three factors, the patterns of PTH and Pi do not directly correlate due to the processes downstream of PTH, such as bone resorption, intestinal absorption, and renal reabsorption.

### 6.4. Interpretations of Circadian Rhythms of Bone-Remodeling-Related Genes and Factors

Circadian rhythms have been described in relation to levels of bone genes, PTH, CT, Ca^2+^, and Pi. However, it has been challenging to directly link these parameters to the local activity levels of bone formation and resorption. It is not possible to definitively state that bone formation or resorption peaks at a certain time of day based on these parameters. This is because we do not know the local levels of the direct effectors of bone remodeling, such as ECM proteins, cathepsin K, H^+^, and Cl^−^. Since some of these parameters are difficult to quantify, in vitro models with synchronized cells should be explored for further investigation.

**Table 4:** Circadian rhythms of serum and plasma concentrations of human PTH, Ca^2+^, and phosphate, and rat CT. Peak and nadir times are described using the 24 h clock. Abbreviations are as follows: PTH—parathyroid hormone; CT—calcitonin; M—men; W—women; and ZT—zeitgeber time.

Hormone and Mineral	Number and Sex of Subjects	Age (Years Old)	Peak Time	Nadir Time	Reference
PTH	8 M and 2 W	22–32	2–4	8–20	[91]
PTH	14 M and 14 W	>65	18–20	10	[72]
PTH	9 M	17–30	1–3 and 19	10–15	[92]
PTH	11 M	20–25	3 and 17	10 and 21	[93]
PTH	6 M and 12 W	28–71	0–4	12–14	[64]
CT	Rat	2 months old	ZT4 and ZT16	ZT0 and ZT8	[94]
Ca^2+^	8 M and 2 W	22–32	20	2–4	[91]
Ca^2+^	14 M and 14 W	>65	12 and 22	4	[72]
Ca^2+^	9 M	17–30	10	16	[92]
Phosphate	8 M and 2 W	22–32	2–4	8–10	[91]
Phosphate	9 M	17–30	3–4	10	[92]

## 7. Phenotypes of *Bmal1* Knockout Mice

The last section focuses on the phenotypic analyses of knockout (KO) mice, targeting circadian regulator genes. It is customary to include both germline KO and bone-specific KO mice in review articles on circadian bone research [14,16,95,96]. However, the following discussion is limited to bone-specific KO mice, since germline KO alters the competing activities of osteoblasts and osteoclasts, in addition to having systemic effects. Only *Baml1* has been used as a target gene in these inducible KO models because conditional *Bmal1* KO mice are widely distributed and *Bmal1* KO makes mice arrhythmic [97], unlike other circadian regulators with multiple orthologs.

### 7.1. Osteoblast-Specific Bmal1 KO Mice

Two groups have published the phenotypes of osteoblast-specific *Bmal1* KO mice (Table 5). In the first study, Takarada et al. found reduced bone mass in the *Osx* promoter-driven osteoblast-specific *Bmal1* KO mice at 8 weeks old [98]. More specifically, they found decreased bone mass, cortical volume, trabecular number, and trabecular thickness in the tibia as well as decreased bone volume in the vertebrae. Another osteoblast-specific *Bmal1* KO created with the *Col1a1* promoter-driven Cre recombinase also demonstrated reduced bone mineralization in the tibia and femur, but the cortical bones were not affected. The authors linked these findings to increased vitamin D-induced *Rankl* expression by KO osteoblasts, resulting in the promotion of osteoclastogenesis and bone resorption. Thus, *Bmal1* in osteoblasts appears to indirectly inhibit bone resorption by suppressing the RANKL axis. The exact circadian timing of these events is not clear. However, given the expression pattern of *Bmal1* mRNA (peak at ZT20 and nadir at ZT14) and the lag period for translation, the BMAL1-based mechanism is perhaps most active during the early light hours of the day. The authors also created osteocyte-specific *Bmal1* KO mice, but they did not show decreased bone mass in the vertebrae (tibia phenotypes were not mentioned) despite an abundant secretion of RANKL by osteocytes, suggesting differentiation stage-specific consequences of *Bmal1* KO in the osteoblast lineage.

The second study, reported by Qian et al., revealed more nuanced results with postnatal *Bmal1* KO induced with the tamoxifen-inducible *Col1a1* promoter [99]. When *Bmal1* depletion was induced on day 5 after birth, adult mice at 8 weeks old demonstrated an increase in bone volume, trabecular number and thickness, and bone mineral density in the femur. This result appears to contradict the study by Takarada et al. discussed above [98], although a direct comparison was not feasible due to the differences in the measured bones and parameters. The increased mass in the trabecular bone was more prominent in 25-week-old KO mice. In contrast, femoral cortical thickness and cortical area were not affected by KO at 8 weeks old, which was consistent with the Takarada study. However, they were decreased by 25 weeks old, demonstrating the bone-region- and age-dependent effects of the KO. The authors went on to show that *Bmal1* KO upregulated the BMP2-SMAD1 (suppressor of mothers against decapentaplegic 1) signaling pathway, one of the main drivers for osteoblast differentiation, as an underlying mechanism for the increased bone mass in the trabecular bone. This result was consistent with the known circadian expression of the BMP2 pathway genes [100]. In contrast, Samsa et al. reported that *Bmal1* KO inhibited the osteoblast differentiation of mesenchymal stem cells in vitro [101], which was inconsistent with the BMP2-based promotion of osteoblastogenesis reported by Qian et al. Collectively, *Bmal1* does control osteoblasts, but its detailed mechanisms appear to be complex and context-dependent.

### 7.2. Osteoclast-Specific Bmal1 KO Mice

The phenotypic analyses of osteoclast-specific *Bmal1* KO mice have been contradictory in a more direct manner, despite applying the same *Ctsk* promoter-Cre driver mice. While Xu et al. detected increased bone mass in the femur and vertebrae, accompanied by diminished osteoclast differentiation in vitro [82], Tsang et al. did not detect any differences in bone mass in the femur or osteoclast differentiation in vitro [102]. The reasons for the discrepancy remain unknown, as Tsang et al. discussed in their paper.

### 7.3. Comments on the KO-Mouse-Based Studies

The exact roles of BMAL1 in osteoblasts and osteoclasts have not been settled. In addition, virtually nothing is known about the roles—if any—of other circadian core regulators, such as CRY, PER, RORs, and Rev-erbs. However, the KO-based approach should provide the most direct connection between circadian rhythms and bone remodeling at the molecular level. Recent advances in spatial multi-omics approaches could support such studies, as discussed below.

**Table 5:** Phenotypes of bone-specific *Bmal1* knockout mice.

Promoter Driving *Cre*	Bone Mass	Other Notable Findings	Reference
*Osx* promoter (osteoblasts)	Decreased in tibial trabeculae and cortex at 8 weeks old	BMAL1 inhibits RANKL expression in osteoblasts	[98]
*Col1a1* promoter (osteoblasts)	Decreased in the tibial trabeculae but not in the cortex at 8 weeks old		[98]
*Dmp1* promoter (osteocytes)	Normal in vertebrae; not described in tibia		[98]
Tamoxifen-inducible *Col1a1* promoter (osteoblasts)	Increased in the femoral trabeculae but not in the cortex at 8 weeks old	BMAL1 inhibited BMP2-SMAD1 pathway	[99]
*Ctsk* promoter (osteoclasts)	Increased in femur and vertebrae	Decreased osteoclast differentiation in vitro	[82]
*Ctsk* promoter (osteoclasts)	Normal in femur	Normal osteoclast differentiation in vitro	[102]

## 8. Significance and Future Perspectives

This review article highlights the knowledge gap between what we know about the molecular details of the functions of bone cells (Section 2 and Section 3 above) and what has been shown to link circadian rhythms and bone cells (Section 5, Section 6 and Section 7). For example, we do not know whether and how the downstream genes of the master regulators RUNX2 and NFATC1 are regulated by circadian rhythms. We also do not know whether the RANKL–RANK axis, M-CSF pathway, and other crosstalk mechanisms are under circadian control. The same is true for the direct parameters of bone formation and resorption, such as the secretion of ECM proteins, the formation of hydroxyapatite, and osteolysis via cathepsin K, H^+^, and Cl^−^. These specific questions lead to a more general question of whether the differentiation of bone cells progresses in an on–off manner (e.g., only active in a specific time window), or is a linear and continuous process. The evidence discussed above favors the on–off hypothesis, but its biological advantage, if any, is even more obscure. These questions need to be addressed heavily relying on in vivo models, mainly mouse models. Fortunately, the recent emergence of spatial transcriptomic approaches at single-cell resolution could revolutionize the field by providing the answers to these heretofore challenging questions (see [103] as an example). These new techniques not only preserve the bone microenvironment, but also reveal position-dependent variations in the biology of bone cells.

Bone remodeling is important for injuries and many diseases, such as fractures, osteoporosis, genetic disorders, bone metastasis, and arthritis. The research on the circadian regulation of bone remodeling is significant not only to determine the correct time window for chronotherapy, but also to identify the novel regulatory mechanisms underlying bone remodeling, using circadian core regulators as starting points.

## Abbreviations

ALP	alkaline phosphatase
*Bmal1*	brain and muscle ARNT-like 1
BMP	bone morphogenetic protein
BMU	basic multicellular unit
BP	β-propeller
BTM	bone turnover marker
CCG	clock-controlled gene
*Clock*	circadian locomotor output cycles protein kaput
*Cry*	cryptochrome
CT	calcitonin
*Ctsk*	cathepsin K
CTX	crosslinked C-terminal telopeptide of type I
DKK1	dickkopf WNT signaling pathway inhibitor 1
ECM	extracellular matrix
FGF	fibroblast growth factor
GIP	glucose-dependent insulinotropic peptide
GLP-1	glucagon-like peptide 1
KO	knockout
LRP5	lipoprotein receptor-related protein 5
M-CSF	macrophage colony-stimulating factor
MMP	matrix metalloproteinase
MSC	mesenchymal stem cell
*Nfatc1*	nuclear factor of activated T cells 1
NF-κB	nuclear factor κ B subunit 1
NTX	crosslinked N-terminal telopeptide of type I
*Opg*	osteoprotegerin
*Osx*	osterix
*Per*	period
Pi	inorganic phosphate
PINP	procollagen type I N-terminal propeptide
PTH	parathyroid hormone
RANK	receptor activator of nuclear factor κB
RANKL	receptor activator of nuclear factor κB ligand
Rev-erbα	reverse orientation c-erbAα
RORα	retinoic acid receptor-related orphan receptor α
RRE	Rev-erb/ROR response element
*Runx2*	runt-related transcription factor 2
SMAD1	suppressor of mothers against decapentaplegic 1
TGF-β	transforming growth factor β
TNF	tumor necrosis factor
Wnt	wingless-related integration site
ZT	zeitgeber time
